# Pathological modeling of glycogen storage disease type III with CRISPR/Cas9 edited human pluripotent stem cells

**DOI:** 10.3389/fcell.2023.1163427

**Published:** 2023-05-11

**Authors:** Lucille Rossiaud, Pascal Fragner, Elena Barbon, Antoine Gardin, Manon Benabides, Emilie Pellier, Jérémie Cosette, Lina El Kassar, Karine Giraud-Triboult, Xavier Nissan, Giuseppe Ronzitti, Lucile Hoch

**Affiliations:** ^1^ CECS, I-Stem, Corbeil-Essonnes, France; ^2^ INSERM U861, I-Stem, Corbeil-Essonnes, France; ^3^ UEVE U861, I-Stem, Corbeil-Essonnes, France; ^4^ Genethon, Evry, France; ^5^ Université Paris-Saclay, Univ Evry, Inserm, Genethon, Integrare Research Unit UMR_S951, Evry, France

**Keywords:** induced pluripotent stem cell, glycogen storage disease, muscular disorders, skeletal muscle cell, CRISPR/Cas9

## Abstract

**Introduction:** Glycogen storage disease type III (GSDIII) is a rare genetic disease caused by mutations in the *AGL* gene encoding the glycogen debranching enzyme (GDE). The deficiency of this enzyme, involved in cytosolic glycogen degradation, leads to pathological glycogen accumulation in liver, skeletal muscles and heart. Although the disease manifests with hypoglycemia and liver metabolism impairment, the progressive myopathy is the major disease burden in adult GSDIII patients, without any curative treatment currently available.

**Methods:** Here, we combined the self-renewal and differentiation capabilities of human induced pluripotent stem cells (hiPSCs) with cutting edge CRISPR/Cas9 gene editing technology to establish a stable *AGL* knockout cell line and to explore glycogen metabolism in GSDIII.

**Results:** Following skeletal muscle cells differentiation of the edited and control hiPSC lines, our study reports that the insertion of a frameshift mutation in *AGL* gene results in the loss of GDE expression and persistent glycogen accumulation under glucose starvation conditions. Phenotypically, we demonstrated that the edited skeletal muscle cells faithfully recapitulate the phenotype of differentiated skeletal muscle cells of hiPSCs derived from a GSDIII patient. We also demonstrated that treatment with recombinant AAV vectors expressing the human GDE cleared the accumulated glycogen.

**Discussion:** This study describes the first skeletal muscle cell model of GSDIII derived from hiPSCs and establishes a platform to study the mechanisms that contribute to muscle impairments in GSDIII and to assess the therapeutic potential of pharmacological inducers of glycogen degradation or gene therapy approaches.

## Introduction

Glycogen storage diseases (GSD) are rare autosomal recessive disorders caused by mutations in genes encoding individual enzymes of the glycogen metabolism pathway (Adeva-Andany et al., 2016). Among them, glycogen storage disease type III (GSDIII; incidence: 1/100,000) is caused by mutations in the *AGL* gene encoding the glycogen debranching enzyme (GDE) which catalyzes the degradation of glycogen (Dagli et al., 1993). The deficiency of this enzyme leads to pathological glycogen accumulation in the liver, skeletal muscles and heart. During childhood, GSDIII patients experience severe fasting hypoglycemia, pronounced hepatomegaly and growth retardation. At adulthood, liver complications such as cirrhosis and/or development of hepatic adenomas and hepatocellular carcinomas can occur (Labrune et al., 1997; Demo et al., 2007; Sentner et al., 2016). Nonetheless, the major disease burden at adulthood is the development of a progressive and severe myopathy with generalized skeletal muscle weakness, exercise intolerance and loss of ambulation. In muscles, glycogen accumulates throughout muscular fibers in multiple foci within the sarcoplasm, impairing muscle function (Laforêt et al., 2019). In addition to skeletal muscle impairments, most adult GSDIII patients have cardiac muscle involvement that can lead in 15% of patients to cardiomyopathy (Sentner et al., 2016). To date, no curative treatment is available for GSDIII. Patients require a strict diet including frequent meals, uncooked cornstarch or continuous enteral feeding to avoid recurrent hypoglycemia. More recently, high-protein diets including ketogenic and modified Atkins diet, were reported to be beneficial in reducing or stabilizing skeletal and cardiac muscle manifestations in some patients (Dagli et al., 2009; Valayannopoulos et al., 2011; Mayorandan et al., 2014; Olgac et al., 2020). Nevertheless, GSDIII and especially its muscle involvement remains an unmet medical need.

Several studies using primary skeletal muscle cells from GSDIII patient biopsies or GSDIII animal models have investigated the pathophysiology of the disease and explored therapeutics for GSDIII. Different strategies, including enzyme replacement therapy (ERT) with the recombinant human enzyme acid alpha-glucosidase (GAA) which induces lysosomal glycogen degradation (Sun et al., 2013), a pharmacological approach based on rapamycin treatment (Yi et al., 2014) or RNA interference therapy through silencing of the liver glycogen synthase *GYS2* gene (Pursell et al., 2018), have demonstrated positive effects on glycogen content in muscle and/or liver cells. More recently, promising recombinant adeno-associated virus (AAV)-based gene therapy approaches demonstrated the feasibility of GDE expression rescue and reduced glycogen content in muscle and liver of a GSDIII mouse model. Although the size of the human *AGL* gene is larger than the size limit of the entire AAV genome, a proof of concept of human *AGL* gene replacement was demonstrated using a dual AAV vector approach (Vidal et al., 2018). More recently, the use of a recombinant AAV vector encoding a smaller bacterial debranching enzyme also corrected glycogen content but was associated with significant immune response toward the bacterial transgene (Lim et al., 2020; Lim et al., 2022). Although these studies have shown promising results, they highlighted the complexity of having a simultaneous and long-term positive impact on muscle and liver phenotypes, demonstrating that efforts are needed to achieve an effective treatment for GSDIII, particularly in muscle.

To better characterize the mechanisms involved in GSDIII muscle pathophysiology and to accelerate the evaluation of therapeutics, we developed an *in vitro* skeletal muscle model of GSDIII derived from human induced pluripotent stem cells (hiPSCs) edited by CRISPR/Cas9 technology. Pluripotent stem cells have unique self-renewal and differentiation capabilities, making them an unlimited biological resource for disease modeling applications and development of new therapies (Karagiannis et al., 2019). Since the discovery of human embryonic stem cells (hESCs) in 1998 (Thomson et al., 1998) and hiPSCs in 2006 (Takahashi and Yamanaka, 2006), effective differentiation protocols for an increasing number of cell types have been reported (Karagiannis et al., 2019). A large number of studies, including from our own group, have highlighted the use of hiPSCs for the pathological modeling of muscular diseases of genetic origin (Tedesco et al., 2012; Tanaka et al., 2013; Shoji et al., 2015; Wu et al., 2017; El-Battrawy et al., 2018; Mateos-Aierdi et al., 2021; Mérien et al., 2021; Bruge et al., 2022). In this study, we first established a stable *AGL* knockout hiPSC line using CRISPR/Cas9 gene editing technology (GSDIII^CRISPR^). Then, we used a robust protocol of differentiation (Caron et al., 2016) to generate pure populations of skeletal myotubes (skMt) from GSDIII^CRISPR^ hiPSCs. Characterization of the cells revealed no GDE expression and persistent glycogen accumulation under glucose starvation conditions. Phenotypically, we demonstrated that GSDIII^CRISPR^ skMt faithfully resembled those differentiated from GSDIII patient-derived hiPSCs. We also demonstrated that glycogen content was restored in GSDIII^CRISPR^ skMt by treatment with recombinant AAV vectors expressing the human GDE, validating the interest of this new *in vitro* GSDIII skeletal muscle model to explore glycogen metabolism for therapeutic purposes.

## Materials and methods

### Cell lines

The CTRL1 hiPSC line is a commercial line provided by Phenocell (Grasse, France), the CTRL2 hiPSC line was reprogrammed from control fibroblasts provided by the Coriell Cell Repository (GM 1869, Camden, United States) and the GSDIII^patient^ hiPSC line was reprogrammed by Phenocell (Grasse, France) from GSDIII fibroblasts provided by the Coriell Institute (GM00576, Camden, United States).

### Cell culture and differentiation

hiPSCs were maintained and expanded using a single-cell method on matrigel (Corning, United States) -coated culture dishes with StemMACS iPS-Brew XF medium (Miltenyi Biotec, Germany). hiPSCs were differentiated into skeletal muscle cells following a protocol developed by Geneabiocell ^®^ (Caron et al., 2016). Briefly, hiPSCs were seeded in collagen I-coated plates (Biocoat, DB Biosciences, United States) and maintained for 10 days in skeletal muscle induction medium (SKM01, AMSBIO, United Kingdom) with a passage at day seven. Cells were then dissociated with 0.05% trypsin (ThermoFisher Scientific, United States) and seeded once again onto collagen I-coated plates for 7 days in skeletal myoblast medium (SKM02, AMSBIO, United Kingdom) until freezing. SkMb were thawed on collagen I-coated plates in SKM02 medium and incubated at confluence with skeletal muscle differentiation medium (SKM03, AMSBIO, United Kingdom) for three supplementary days. For the glucose deprivation experiments, skMt were incubated with glucose-free DMEM (ThermoFisher Scientific, United States) supplemented with 10% Fetal Bovine Serum (FBS, Sigma, United States) for five supplementary days prior to the glycogen analysis.

### GSDIII^CRISPR^ hiPSC line generation

We generated the GSDIII^CRISPR^ hiPSC line by CRISPR/Cas9-mediated genome edition. SpCas9 target sequences within the coding exons of *AGL* were determined by CRISPOR (http://crispor.tefor.net/). The sgRNA with the highest predicted efficiency, the lowest number of potential off-targets and having an enzymatic restriction site at the PAM sequence was selected. The selected sgRNA targets the exon 5 of *AGL* and its sequence is: 5′-GGG​GCC​ACT​AGG​GAC​AGG​AT-3′. crRNA and tracrRNA were synthesized by Integrated DNA Technologies^®^. Prior to transfection, we performed the ribonucleoprotein complex formation by combining SpCas9 protein (gift from Jean-Paul Concordet, MNHN–CNRS UMR7196/INSERM U1154) with the sgRNA. Control hiPSCs (CTRL1) were dissociated with StemPro Accutase Cell Dissociation Reagent (Invitrogen, United States). About 100,000 cells were first incubated with the RNP complex and then electroporated using the NEON nucleofector (Invitrogen, United States), with two pulses at 1,200 V, width 20 ms. After transfection, the hiPSCs were collected and plated in StemMACS iPS-Brew XF medium (Miltenyi Biotec, Germany) with Y-27632 rock inhibitor 10 µM (Miltenyi Biotec, Germany) on a matrigel (Corning, United States)-coated culture dish. After 48 h, cells were detached and seeded back to get isolated clones from the pool using limiting dilution cloning in a 96-well plate. After 14 days of expansion, 24 hiPSC clones were harvested for genomic DNA (gDNA) extraction.

### gDNA extraction and PCR

gDNA was extracted from transfected hiPSCs with QIAmp DNA Mini Kit (Qiagen, Germany) according to the manufacturer’s instructions. PCR were performed using Phusion ™ High-Fidelity DNA Polymerase (Thermofisher Scientific, United States) with primers framing the target area of the sgRNA (listed in Supplementary Table S1) and 200 ng of gDNA for 35 cycles of 10 s at 98°C, 30 s at 60°C, and 15 s at 72°C, with a final 5 min extension.

### RFLP assays

Restriction Fragment Length Polymorphism (RFLP) was performed to rapidly estimate the presence of gene editing in the 24 isolated clones. Briefly, 2 μL PCR products were digested by five units of BsrD1 restriction enzyme (New England Biolabs, France) for 1 h at 65°C. Cleaved fragments were then separated by gel electrophoresis. When CRISPR-mediated gene editing was successful, non-homologous end joining-mediated small insertions/deletions abolished the restriction endonuclease recognition site. After restriction digest, 6 clones had completely lost their restriction site. One clone (clone 17) was selected and its corresponding PCR product was sequenced by Sanger DNA sequencing (Genewiz, Germany).

### Off-target analysis

We used the online tool CRISPOR (https://crispor.tefor.net/) to predict 8 potential off-target sites of the sgRNA (Supplementary Table S2). We designed primers listed in Supplementary Table S1 for these sites and used them to amplify these regions by PCR with gDNA from GSDIII^CRISPR^ hiPSCs. PCR products were then sequenced by Sanger DNA sequencing (Genewiz, Germany).

### Flow cytometry

Single-cell suspension of hiPSC was collected after chemical dissociation with accutase (Invitrogen, United States), centrifuged at 900 rpm for 5 min, and resuspended in 2% FBS (Sigma, United States) in cold PBS. Cells were stained with fluorescent dye-conjugated antibodies (listed in Supplementary Table S3) for 30 min on ice and protected from light. Cells were washed in cold PBS before being assessed by a MACSquant analyzer (Miltenyi Biotec, Germany). Data were analyzed with FlowJo Software (BD Biosciences, United States).

### Multiplex fluorescence in situ hybridization (m-FISH) karyotype analysis

Cells were blocked in metaphase with colchicine (Eurobio, France) for 90 min, warmed with a hypotonic solution, and fixed with a Carnoy fixative. A M-FISH 24Xcite probe (MetaSystems, Germany) and ProLong Gold Antifade Mountant with DAPI (ThermoFisher Scientific, United States) were used for m-FISH staining. Seventy metaphases were acquired with Metafer MetaSystems software coupled to an AxioImager Z2 (Zeiss, Germany) microscope equipped with a camera cool cube and ×10 and ×63 objectives. Images were analyzed with Isis software (MetaSystems).

### Quantitative PCR

Total RNAs were isolated using the RNeasy Plus Mini extraction kit (Qiagen, Germany) according to the manufacturer’s instructions. DNase I digestion was performed to degrade DNA in the sample. RNA levels and quality were checked using the NanoDrop technology. A total of 500 ng of RNA was used for reverse transcription using the SuperScript III reverse transcription kit (Invitrogen, United States). Quantitative polymerase chain reaction (qPCR) analysis was performed using a QuantStudio 12 K Flex real-time PCR system (Applied biosystem, United States) and Luminaris Color HiGreen qPCR Master Mix (Thermo Scientific, United States), following the manufacturers’ instructions. Quantification of gene expression was based on the DeltaCt method and normalized on 18S expression (Assay HS_099999). The primers used in this study are reported in Supplementary Table S1.

### Western blot

Whole-cell lysate of control and mutated skMt were collected after 4 days of differentiation. Proteins were extracted with NP40 lysis buffer (Thermo Scientific, United States) supplemented with 1X Proteases Inhibitors (Complete PIC, Roche, Switzerland). Protein concentration was evaluated using the Pierce BCA Protein Assay Kit (Thermo Scientific, United States) and the absorbance was measured at 562 nm using a CLARIOstar^®^ microplate reader (BMG Labtech, Germany). For GDE protein detection, a total of 50 μg of protein was separated using a 4%–15% Criterion™ XT tris-glycine protein gel (BioRad, United States) and then transferred to PVDF membrane (BioRad, United States) with a Trans-Blot Turbo Transfert system (BioRad, United States) following the manufacturer’s instructions. Membrane was blocked in Odyssey blocking buffer (Li-Cor, United States) for 1 h at room temperature and then incubated with primary antibodies (listed in Supplementary Table S3) diluted in blocking buffer at room temperature for 2 h. Washing was carried out three times for 10 min at room temperature with TBS +0.1% Tween 20 (VWR, United States) and the membrane was incubated with appropriate fluorescent secondary antibodies (listed in Supplementary Table S3) in blocking buffer at room temperature for 2 h. Washing was carried out, and proteins were detected by fluorescence (Odyssey, Li-Cor, United States) following the manufacturer’s instructions.

### Immunostaining assay

hiPSCs and skMt were fixed with 4% paraformaldehyde (Euromedex, France) for 10 min at room temperature. After 3 washes in phosphate-buffered saline (PBS), cells were permeabilized with 0.5% Triton X-100 (Sigma, United States) for 10 min and blocked in PBS solution supplemented with 1% bovine serum albumin (BSA, Sigma, United States) for 1 h at room temperature. Cells were stained for specific markers overnight at 4°C using primary antibodies (listed in Supplementary Table S3). After three washes in PBS staining was revealed by appropriate Alexa Fluor secondary antibodies (listed in Supplementary Table S3) in the dark for 1 h at room temperature, and nuclei were visualized with Hoechst solution 1:2000 (Invitrogen, United States). SkMt imaging was carried out with a HCS Navigator™ (Version 6.6.0) software-associated Cell Insight™ CX7 Plateform automated microscope (ThermoFisher Scientific, United States) with a ×20 or ×40 objective. hiPSCs imaging was carried out with a Zen Blue software-associated Observer Z1 epifluorescent microscope (Zeiss, Germany) with a ×20 objective.

### Periodic Acid Schiff (PAS) staining

PAS staining was performed with the PAS Staining Kit (Sigma-Aldrich, United States) following the manufacturer’s instructions. Briefly, cells were fixed with 4% paraformaldehyde for 10 min at room temperature. After two washes in PBS, skMt were treated with Periodic Acid Solution for 5 min at room temperature. After three washes in tap water, cells were treated with Schiff’s reagent for 15 min at room temperature. Finally, after four washes in tap water, staining was visualized using an EVOS XL Core microscope (Invitrogen, United States). Images were processed and analyzed using FIJI custom-made scripts (Schindelin et al., 2012). First, colors were split and only the green channel was kept as it was the most contrasted one. Then, images were manually thresholded into binary images where PAS signal was in black and background in white. The same threshold was used for all the images. The quantification of PAS staining was obtained using this formula: (Area of PAS staining/Total area of image) ×100, giving then a percentage of PAS staining within the image.

### Measurement of glycogen content

Glycogen content was measured using the Glycogen-Glo assay (Promega, United States) following the manufacturer’s instructions. Briefly, skMt were lysed using an HCl acidic solution to aid in cell disruption, inactivation of endogenous enzymes and degradation of endogenous NADH. Glycogen was then digested into glucose by incubation with glucoamylase enzyme for 1 h at room temperature. The resulting glucose was then measured using glucose dehydrogenase in conjunction with a bioluminescent NADH detection reagent incubated for 1 h 30 min at room temperature. The result was a light signal proportional to the starting glycogen concentration in skMt. Luminescence was read using a CLARIOstar^®^ microplate reader (BMG Labtech, Germany). Raw data related to glycogen content were normalized on the number of viable cells measured by luminescence using CellTiter-Glo assay (Promega, United States).

### Measurement of GDE enzymatic activity

GDE activity was performed as previously described (Vidal et al., 2018). Briefly, cell lysates were incubated à 37°C with limit dextrin for 6 h and the glucose released by GDE was measured using the glucose assay kit (Sigma, United States) following the manufacturer’s instructions. An incubation without limit dextrin was performed in parallel to exclude the absence of glucose in the background. Enzymatic activity was expressed in µmoles of released glucose per Gram of total protein and per minute.

### Recombinant AAV transduction

Recombinant AAV vectors containing a GDE expression cassette were generated using an adenovirus-free transient transfection method (Matsushita et al., 1998) and purified as described earlier (Collaud et al., 2019). The capsid used was the published LK03 capsid (Lisowski et al., 2014), which has been shown to efficiently transduced hiPSCs (Westhaus et al., 2020). The GDE expression cassette contained a truncated version of the cytomegalovirus (CMV) promoter (nucleotides 175050_175400 of the CMV genome, NC_006273), the human full-length codon-optimized GDE cDNA (Mingozzi et al., 2018; Vidal et al., 2018) and a short poly-A signal of 58 bp (Potter et al., 2021). The cassette was flanked by inverted terminal repeats of AAV serotype 2 for vector packaging. The resulting expression cassette was oversized and was produced at low yield although sufficient for *in vitro* use. Titers of the AAV vector stocks [8 × 10^11^ vector genome (vg)/mL] were determined using a real-time qPCR using primers for the GDE transgene listed in Supplementary Table S1. SkMb were transduced 2 days after seeding in SKM02 (AMSBIO, United Kingdom) with AAV vectors expressing the human GDE at a multiplicity of infection (MOI) of 75,000. At confluence, medium was changed with SKM03 (AMSBIO, United Kingdom) for 3 days. Differentiated skMt were then incubated with glucose-free DMEM (ThermoFisher Scientific, United States) supplemented with 10% FBS (Sigma, United States) for five supplementary days prior to the glycogen analysis.

### Statistical analysis

Data are presented as means ± SD (*n* = 3) of a representative experiment over three independent experiments. Statistical analysis was performed using one-way ANOVA and the Bonferroni’s multiple comparison test. Statistical significance was considered for ***p ≤ 0.001, **p ≤ 0.01, *p ≤ 0.05. Histograms were performed using GraphPad Prism (v8.4.3).

## Results

### Generation of the GSDIII^CRISPR^ hiPSC line

**FIGURE 1:**
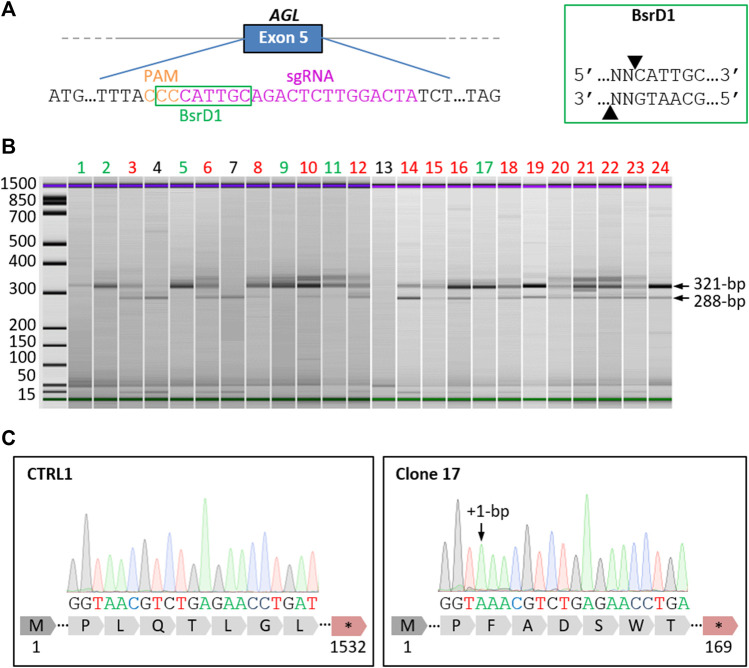
Generation of a homozygous *AGL* knockout hiPSC line by CRISPR/Cas9. **(A)** Schematic representation of the *AGL* gene with an extended part of the exon 5 sequence showing the location of the selected specific guide RNA (sgRNA, magenta) and Protospacer Adjacent Motif (PAM, orange). The recognition site of the restriction enzyme BsrD1 is framed in green. The cut-off site of BsrD1 is indicated on the right box. **(B)** Electrophoresis analysis of PCR products from the 24 isolated clones digested with BsrD1 restriction enzyme. When CRISPR gene editing was successful, the restriction endonuclease recognition site was abolished, resulting in the detection of 321-bp fragments. Six clones had totally lost Bsrd1 restriction site (green), fifteen clones had partially lost BsrD1 restriction site (red) and three clones had not lost BsrD1 restriction site (black). **(C)** Sequencing of PCR products revealed a homozygous 1-bp insertion in the edited *AGL* gene of clone 17 compared to CTRL1, causing a frameshift mutation in *AGL* and generating a premature stop codon at amino acid 169 of GDE.

To generate the GSDIII^CRISPR^ hiPSC line, we used the CRISPR/Cas9 gene editing technology. The classical mechanism of non-homologous end joining which mediates DNA double-strand breaks repair was exploited to generate small insertions/deletions (INDELS) that could lead to frameshift mutations and premature stop codon apparitions in the *AGL* gene. The specific guide RNA (sgRNA) was designed to target exon 5 of *AGL* and to include an enzymatic restriction site at the protospacer adjacent motif (PAM) sequence (Figure 1A). Briefly, the sgRNA was complexed with SpCas9 protein to perform ribonucleoprotein transfection of dissociated control hiPSCs (CTRL1). Restriction fragment length polymorphism analysis of the pool of transfected cells revealed a percentage of 80% of edited cells (data not shown). Edited hiPSCs were then isolated from the pool of transfected cells using limiting dilution cloning. The selection of gene-edited clones was based on a two-step selection process. Thanks to the use of a sgRNA targeting a sequence within a restriction endonuclease site at the PAM level (Figure 1A), restriction fragment length polymorphism was analyzed to select gene-edited clones, based on the total loss of the restriction site and the identification of a fragment of 321-bp. The second selection was based on Sanger DNA sequencing to select clones with INDELS that have generated stop codons. Of the twenty-four clones analyzed, six clones showed a single 321-bp fragment (Figure 1B) demonstrating complete loss of the BsrD1 restriction site and generation of homozygous INDELS in these clones. Fifteen clones showed both 321-bp and 288-bp fragments demonstrating partial loss of the BsrD1 restriction site and generation of heterozygous INDELS. Three clones showed a single 288-bp fragment indicating no loss of the BsrD1 restriction site and no gene editing (Figure 1B). Among the clones having totally lost their BsrD1 restriction site, one clone (clone 17) was selected (Figure 1C). Sequencing revealed a homozygous 1-bp insertion in the edited *AGL* gene of clone 17 compared to CTRL1 hiPSCs, causing a frameshift mutation in *AGL* and generating a premature stop codon at amino acid 169 of GDE (Figure 1C). To determine off-target activity of the sgRNA, we analyzed eight potential off-target sites (Supplementary Table S2). Sequencing revealed no differences compared to control sequences, demonstrating the absence of mutagenesis in these eight potential off-target sites in GSDIII^CRISPR^ hiPSCs (Supplementary Figure S1).

### Characterization of GSDIII^CRISPR^ hiPSCs pluripotency capacities

**FIGURE 2:**
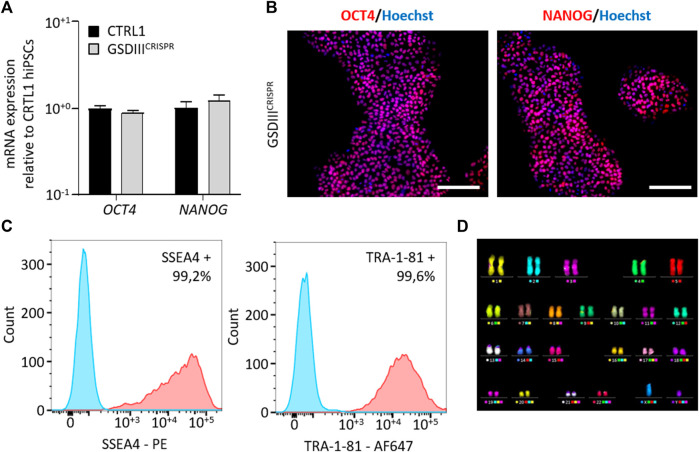
Characterization of the GSDIII^CRISPR^ hiPSCs pluripotency capacities. **(A)** mRNA expression levels of *OCT4* and *NANOG* in CTRL1 and GSDIII^CRISPR^ hiPSC lines measured by qPCR in triplicate. mRNA expression analyses are normalized to CTRL1 hiPSCs. **(B)** Characterization of the expression of OCT4 and NANOG by immunostaining in GSDIII^CRISPR^ hiPSCs. Nuclei are labeled by Hoechst staining (blue). Scale bar = 200 µm. **(C)** Characterization of the expression of SSEA4 and TRA1-81 by flow cytometry in GSDIII^CRISPR^ hiPSCs. The Fluorescence Minus One control condition is represented in blue and the marked condition is represented in red. **(D)** Karyotyping analysis of the GSDIII^CRISPR^ hiPSC line.

A full quality control of the pluripotency capacities of GSDIII^CRISPR^ hiPSCs was realized. Quantitative PCR analysis revealed no significant differences in the mRNA expression levels of the pluripotent markers *OCT4* and *NANOG* between CTRL1 and GSDIII^CRISPR^ hiPSC lines (Figure 2A). Immunostaining analysis indicated that GSDIII^CRISPR^ hiPSCs expressed the pluripotent markers OCT4 and NANOG at the protein level (Figure 2B). The pluripotency status of the GSDIII^CRISPR^ hiPSC line was also validated by measuring the expression of SSEA4 and TRA1-81 using flow cytometry (Figure 2C). Finally, chromosomal stability was analyzed using the multiplex fluorescence *in situ* hybridization (m-FISH) technique, revealing the absence of significant abnormalities in GSDIII^CRISPR^ hiPSC line (Figure 2D).

### Differentiation of GSDIII^CRISPR^ and isogenic CTRL1 hiPSCs into skeletal muscle cells

**FIGURE 3:**
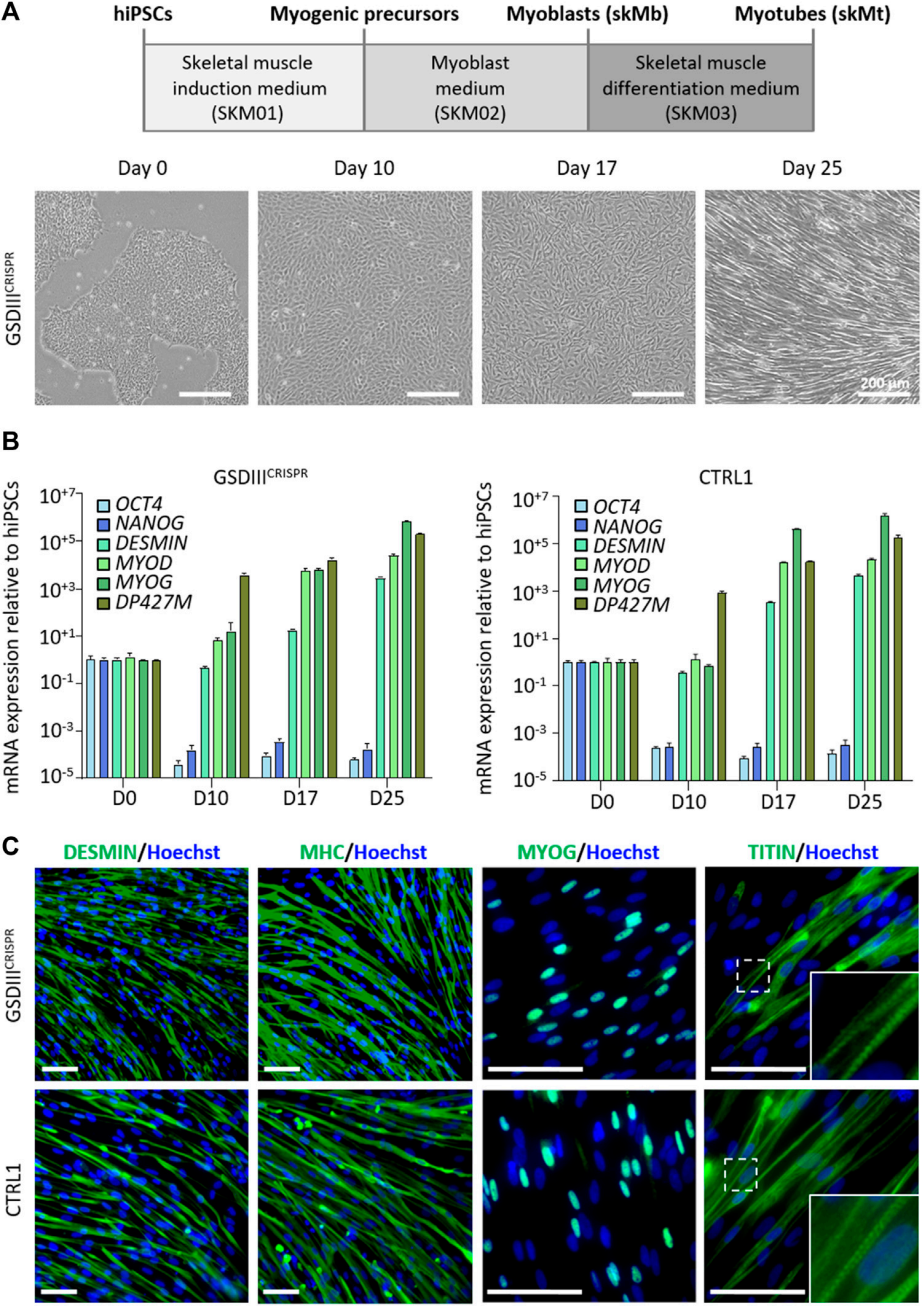
Skeletal myogenic differentiation of GSDIII^CRISPR^ and isogenic CTRL1 hiPSCs. **(A)** Schematic representation of the skeletal myogenic differentiation protocol and phase contrast microscopic images of cell morphology during differentiation. Scale bar = 200 µm. **(B)** mRNA expression levels of pluripotency markers (*OCT4*, *NANOG*) and myogenic markers (*DESMIN*, *MYOD*, *MYOG*, *DP427M*) measured by qPCR in triplicate at day 0, 10, 17, and 25 of differentiation of GSDIII^CRISPR^ and CTRL1 hiPSCs. mRNA expression analyses are normalized to hiPSCs at day 0. **(C)** Characterization of the expression of desmin, myosin heavy chain (MHC), myogenin (MYOG) and titin by immunostaining in GSDIII^CRISPR^ and CTRL1 skMt (D25). Nuclei are labeled by Hoechst staining (blue). White boxes are magnifications of the titin staining. Scale bar = 50 µm.

Skeletal myogenic differentiation of the GSDIII^CRISPR^ and the isogenic CTRL1 hiPSC lines were induced using a three-step protocol of differentiation (Caron et al., 2016). Briefly, undifferentiated hiPSCs were incubated during 10 days in SKM01 medium to initiate the differentiation into myogenic precursors before their maturation into skeletal myoblasts (skMb) in SKM02 medium and their terminal differentiation into skMt in SKM03 medium (Figure 3A). Quantitative PCR analysis revealed decreased mRNA expression levels of the pluripotency markers *OCT4* and *NANOG* in skMb and skMt compared to undifferentiated hiPSCs and increased mRNA expression levels of the myogenic markers *DESMIN*, *MYOD*, *MYOG*, and *DP427M* in skMb and skMt (Figure 3B). Comparative profiles of mRNA expression levels were observed on GSDIII^CRISPR^ and CTRL1 cells, demonstrating similar myogenic differentiation efficiency of both hiPSC lines (Figure 3B). Protein analysis by immunostaining confirmed the expression of skMt markers such as desmin, myosin heavy chain (MHC), myogenin (MYOG) and the striated pattern of titin in terminally differentiated GSDIII^CRISPR^ and CTRL1 skMt (Figure 3C).

### Glycogen debranching enzyme deficiency and glycogen accumulation in skMt derived from mutated hiPSCs

**FIGURE 4:**
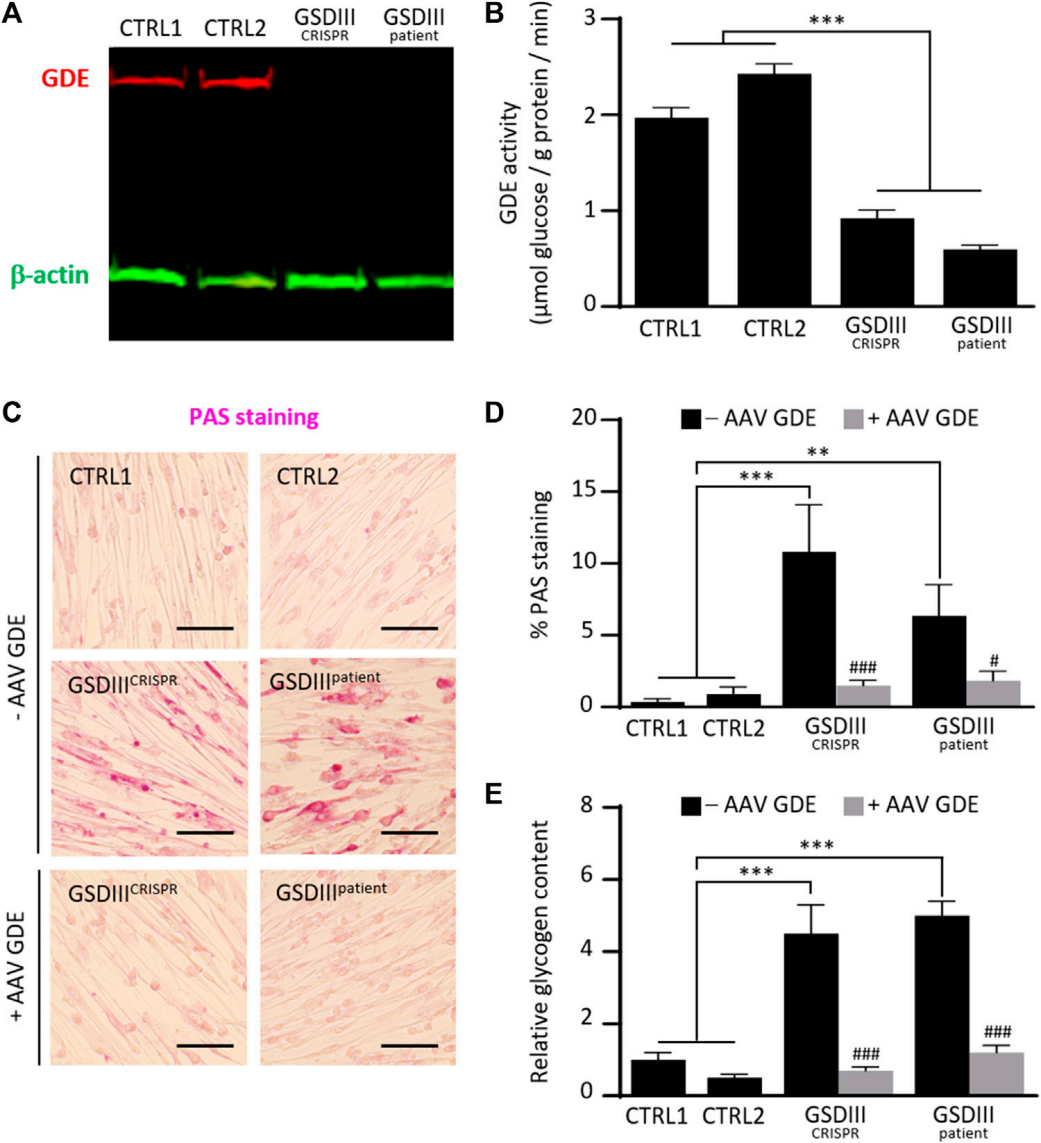
Phenotypical characterization of control and mutated skMt derived from hiPSCs. **(A)** Immunoblot analysis of GDE expression (red) in control and mutated skMt derived from hiPSCs. *β*-actin (green) was a loading control. **(B)** GDE activity measured in starved control and mutated skMt, in triplicate. The activity of the enzyme is expressed in µmoles of glucose released per Gram of protein per minute. **(C)** Representative images of Periodic Acid Schiff (PAS) staining of starved control and mutated skMt treated or not during the myogenic differentiation with a recombinant AAV vector expressing the human full-length GDE. Transduction was performed in triplicate at a MOI of 75,000. Scale bar = 200 µm. **(D)** Quantitative analysis of PAS staining in starved control and mutated skMt treated or not during the myogenic differentiation with a recombinant AAV vector expressing the human full-length GDE. PAS staining is expressed as percentage of PAS positive pixels. **(E)** Measurement of glycogen content using photometric assay in starved control and mutated skMt treated or not during the myogenic differentiation with a recombinant AAV vector expressing the human full-length GDE. Transduction was performed in triplicate at a MOI of 75,000. Glycogen content is expressed as the relative amount of glycogen measured on CTRL1 line. Statistical analysis was performed using one-way ANOVA and the Bonferroni’s multiple comparison test. Significance indicated with * vs. CTRLs skMt and # vs. respective non-treated mutated skMt. *** and ###*p* ≤ 0.001, **p ≤ 0.01, #*p* ≤ 0.05.

To investigate the phenotype of skMt derived from the GSDIII^CRISPR^ hiPSC line, we analyzed the expression and activity of the GDE and the glycogen content of GSDIII^CRISPR^ and CTRL1 skMt. Analyses were performed in parallel with additional mutated and control skMt differentiated from hiPSCs. GSDIII^patient^ skMt were differentiated from hiPSCs, generated through the reprogramming of GSDIII patient fibroblasts (GM00576, Coriell Institute), which were characterized and differentiated by the same protocols as the GSDIII^CRISPR^ and CTRL1 hiPSC lines (Supplementary Figures S2, S3). CTRL2 skMt were differentiated from a control hiPSCs characterized previously (Bruge et al., 2022) and differentiated by the same protocol (Supplementary Figure S3). Western blot analysis demonstrated the absence of GDE expression on both mutated skMt compared to controls (Figure 4A). Measurement of the GDE enzymatic activity in presence of limit dextrin revealed a significant decrease in glucose generated by mutated skMt compared to controls confirming reduced GDE activity (Figure 4B). Phenotypically, we observed that, in the presence of glucose in the culture medium, control skMt had similar glycogen content than mutated skMt by Periodic Acid Schiff (PAS) staining and an enzymatic assay (Supplementary Figure S4). We demonstrated that a period of 5 days of glucose deprivation was necessary to clear glycogen in the control skMt and to reveal a significant difference of glycogen content between mutated and control skMt (Figures 4C–E). Finally, to confirm that the glycogen accumulation observed in GSDIII^CRISPR^ and GSDIII^patient^ skMt was caused by the absence of GDE, GSDIII^CRISPR^ and GSDIII^patient^ skMb were treated with a recombinant AAV vector expressing the human full-length GDE during myogenic differentiation to rescue the expression of the protein. Western blot analysis demonstrated a restoration of GDE expression in treated GSDIII^CRISPR^ skMt, accounting for around 50% of the GDE expression observed in healthy CTRL1 skMt (Supplementary Figure S5). Glycogen analysis, by PAS staining and the enzymatic assay, revealed a significant decrease of glycogen content in treated GSDIII^CRISPR^ and GSDIII^patient^ skMt, suggesting that the restoration of a functional GDE activity was able to clear the glycogen accumulated in the two cell lines. (Figures 4C–E).

## Discussion

The main result of this study is the demonstration that skMt derived from GSDIII^CRISPR^ hiPSC can be used to recapitulate the main molecular feature of GSDIII, i.e., cytosolic glycogen accumulation. In particular, we proved that the GSDIII^CRISPR^ skMt persist to accumulate glycogen under glucose starvation conditions and faithfully resembled the phenotype observed in differentiated skMt of hiPSCs derived from a GSDIII patient. The need of glucose deprivation to observe the phenotype is explained by the composition of the myogenic differentiation medium which contains insulin and glucose. While insulin is necessary for myogenic differentiation, in the presence of extracellular glucose, it also induces glycogen synthesis (Saltiel and Kahn, 2001). We demonstrated that after 5 days of glucose deprivation in the absence of insulin, glycogen was completely degraded in control skMt, while accumulated in GSDIII skMt.

To date, evaluations of potential GSDIII therapies in human skeletal muscle cells have been performed on primary cells obtained from patient’s biopsies (Sun et al., 2013; Yi et al., 2014). Muscle biopsies are invasive and painful procedures with transient primary cell expansion, and the rarity of the GSDIII limits their availability. In order to overcome these technical difficulties, hiPSCs have emerged as a relevant alternative. Indeed, the ease of reprogramming hiPSCs from blood samples or skin biopsies and recent advances in the development of skeletal myogenic differentiation protocols have allowed the generation of human skeletal muscle cell models suitable for the study of rare muscle diseases (Yoshida et al., 2017; Bruge et al., 2022; Guo et al., 2022; Laberthonnière et al., 2022; Ortuño-Costela et al., 2022; Shahriyari et al., 2022). Due to their unique self-renewal capacity, hiPSCs represent a standardized and unlimited source of cells, allowing the production of virtually infinite skeletal muscle cells and opening application for high-throughput drug screening. In this study, we demonstrated that our model can be used to quantitatively evaluate the response of recombinant AAV vectors expressing the human GDE on the glycogen content of the cells. Through this experiment, we demonstrated that our cellular model is relevant to study the efficacy of new therapies for GSDIII. Since no molecular target has been identified for GSDIII, a phenotypic screening approach based on the measurement of glycogen content of GSDIII^CRISPR^ skMt represents a valuable strategy to evaluate the therapeutic benefit of gene therapy vectors or pharmacological compounds able to induce glycogen degradation. Indeed, phenotypical approaches have already been applied in drug screening for both common and rare diseases, with the discovery for example, of ivacaftor for cystic fibrosis (Van Goor et al., 2009) or risdiplam for spinal muscular atrophy (Ratni et al., 2018).

Animal models of GSDIII have also been described as good models to study the pathology and evaluate potential treatments. A natural GSDIII canine model (Yi et al., 2012) and different *AGL* knockout mouse models (Liu et al., 2014; Pagliarani et al., 2014; Pursell et al., 2018; Vidal et al., 2018) have been biochemically and histologically characterized. In those models, the lack of GDE expression and the glycogen accumulation in liver and muscle was demonstrated and correlated with hepatic impairments and muscle damage. Although animal models have become increasingly important for the development and evaluation of therapeutics in the real-organism environment and context. These models have limitations such as long generation time, maintenance costs, the impossibility to perform high-throughput screening and sometimes problems of transposition to humans. The use of hiPSCs overcomes many of these limitations and is an interesting strategy for identifying a treatment in the first instance. Even if the skeletal muscle cells derived from hiPSCs could be criticized on their degree of maturity, displaying embryonic/foetal phenotype (Mournetas et al., 2021), differentiation protocols are becoming more complex in order to obtain more mature cells, in particular through 3D cultures in hydrogels (Maffioletti et al., 2018; Rao et al., 2018; Shahriyari et al., 2022; Smith et al., 2022). In this study, we demonstrated that our 2D differentiated skeletal muscle model was sufficiently mature to recapitulate the molecular phenotype of GSDIII.

Since GSDIII is also a disease that affects the liver and the heart, the generation of the GSDIII^CRISPR^ hiPSC line open to the possibility to differentiate the cells through available differentiation protocols into hepatocytes (Si-Tayeb et al., 2016; Messina et al., 2022) or cardiomyocytes (Zhang et al., 2009; Zwi et al., 2009; Goldfracht et al., 2019) to model GSDIII liver and heart deficiencies. Such new cellular models will improve the understanding of the pathophysiology of GSDIII while providing a cellular model to test the efficacy of small molecules or gene therapies identified in the skeletal muscle cellular model. Finally, another advantage of having generated a hiPSC line genetically modified on *AGL* gene from a healthy hiPSC line using CRISPR/Cas9 technology is that we obtained two isogenic lines that can be easily compared. Indeed, since these two lines have the same genetic background, after differentiation into skeletal muscle cells, a comparative transcriptomic analysis between the two lines may simplify the identification of differentially expressed genes or signaling pathways that specifically contribute to muscle impairments in GSDIII. This novel cellular model therefore represents a valuable tool to study the muscular pathophysiology of the disease. In addition, an enrichment analysis may allow the identification of new potential pathological biomarkers of GSDIII that can then be monitored to evaluate the efficacy of GSDIII therapies. Altogether, our results demonstrate that the newly generated GSDIII^CRISPR^ hiPSC model provides a platform to study the pathological molecular mechanisms of GSDIII and to evaluate the *in vitro* efficacy of future GSDIII therapies.

## Data Availability

The original contributions presented in the study are included in the article/Supplementary Material, further inquiries can be directed to the corresponding authors.
