# Supplementary material for: Pathological modeling of glycogen storage disease type III with CRISPR/Cas9 edited human pluripotent stem cells
Source: Front Cell Dev Biol. 2023 May 11;11:1163427. doi: 10.3389/fcell.2023.1163427 (PMC10213880; doi:10.3389/fcell.2023.1163427)
Supplement: Supplementary file 1 [file DataSheet1.PDF]

Supplementary Figure S1

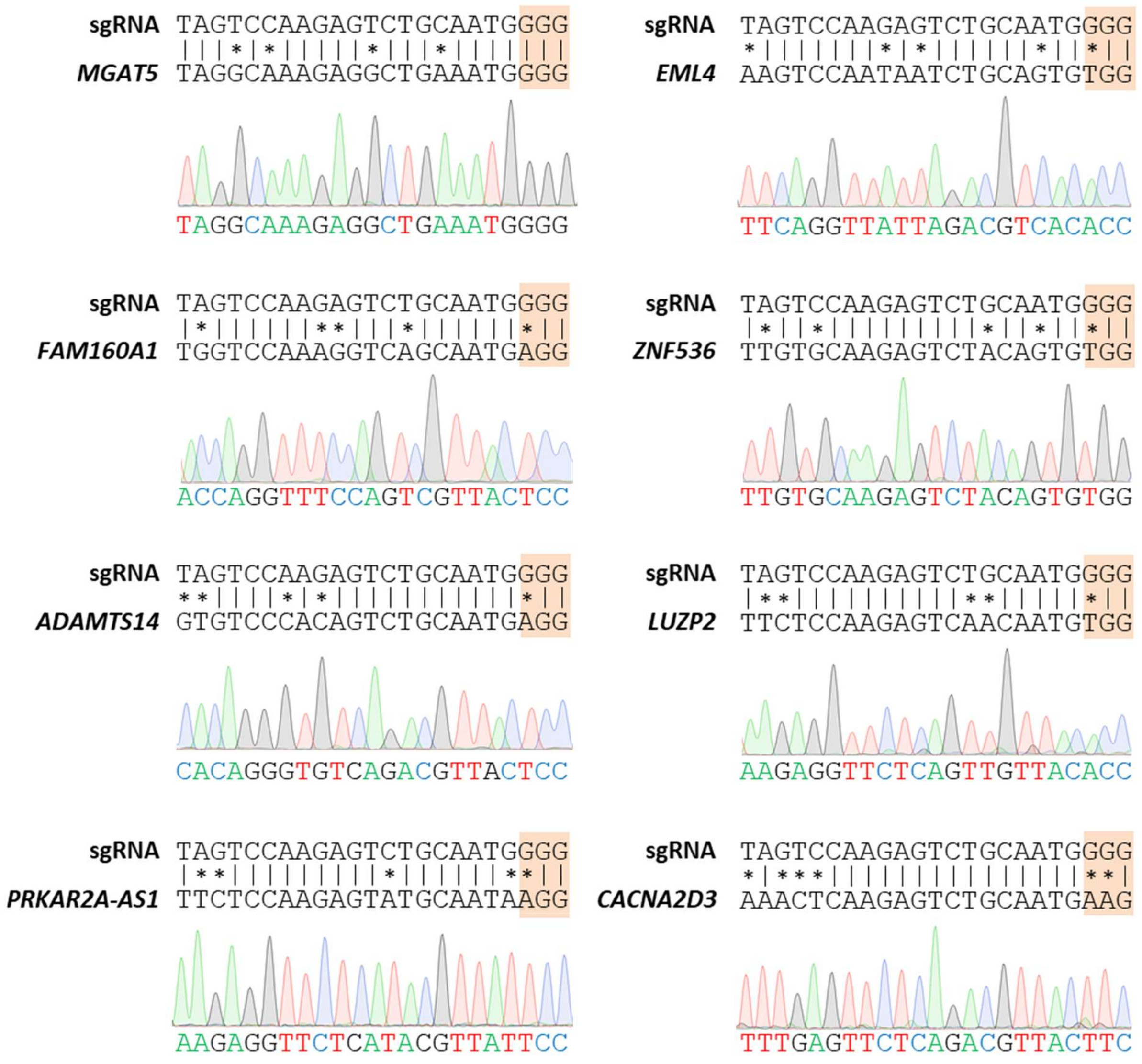

Supplementary Figure S1. SgRNA off-targets analysis. Schematic representation of the sequence alignment of the selected specific guide RNA (sgRNA) and 8 potential off-target sites. The location of mismatched nucleotides (*) and Protospacer Adjacent Motif (orange) are indicated. Primers for these sites were designed (Supplementary Table S1) and used to perform PCR with gDNA from GSDIII^{CRISPR} hiPSCs. Sequencing of PCR products revealed no differences compared to control sequences, demonstrating the absence of mutagenesis in these 8 potential off-target sites in edited hiPSCs.

Supplementary Figure S2

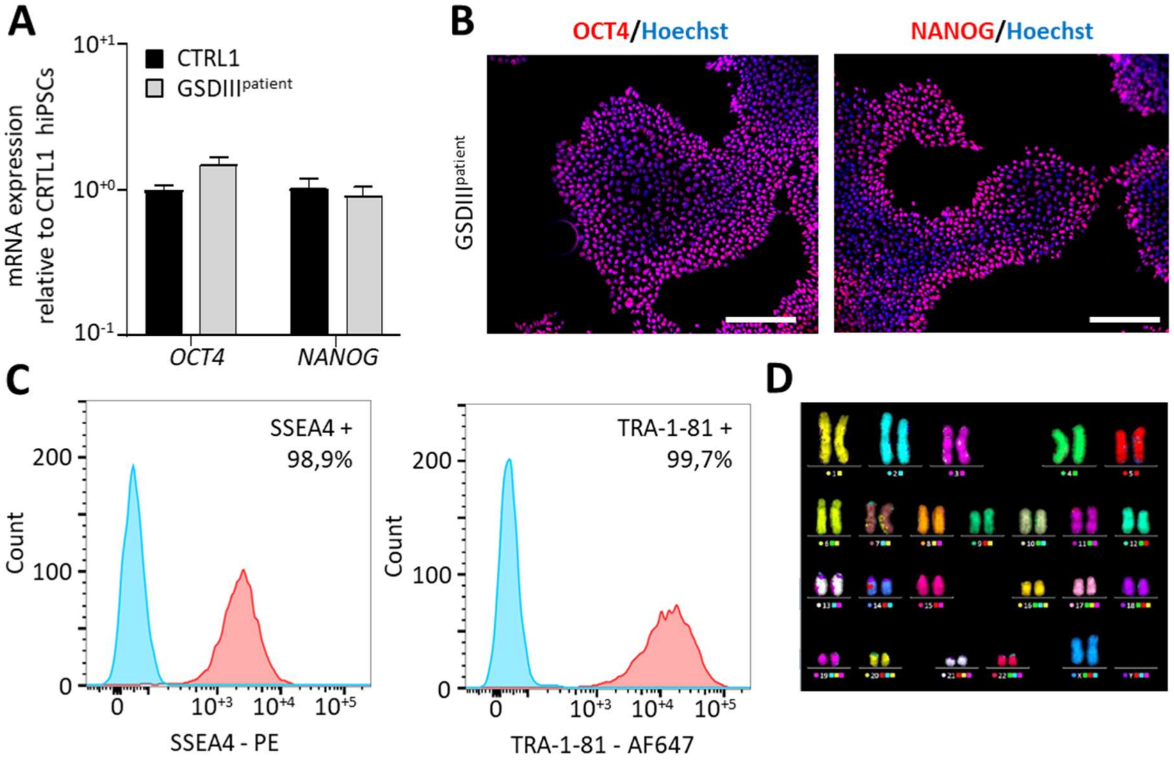

Supplementary Figure S2. Characterization of the GSDIII^{patient} hiPSCs pluripotency capacities. (A) mRNA expression levels of *OCT4* and *NANOG* in control and GSDIII^{patient} hiPSC lines measured by qPCR in triplicate. mRNA expression analyses are normalized to CTRL1 hiPSCs. (B) Characterization of the expression of OCT4 and NANOG by immunostaining in GSDIII^{patient} hiPSCs. Nuclei are labeled by Hoescht staining (blue). Scale bar = 200 µm. (C) Characterization of the expression of SSEA4 and TRA-1-81 by flow cytometry in GSDIII^{patient} hiPSCs. The Fluorescence Minus One control condition is represented in blue and the marked condition is represented in red. (D) Karyotyping analysis of the GSDIII^{patient} hiPSC line.

Supplementary Figure S3

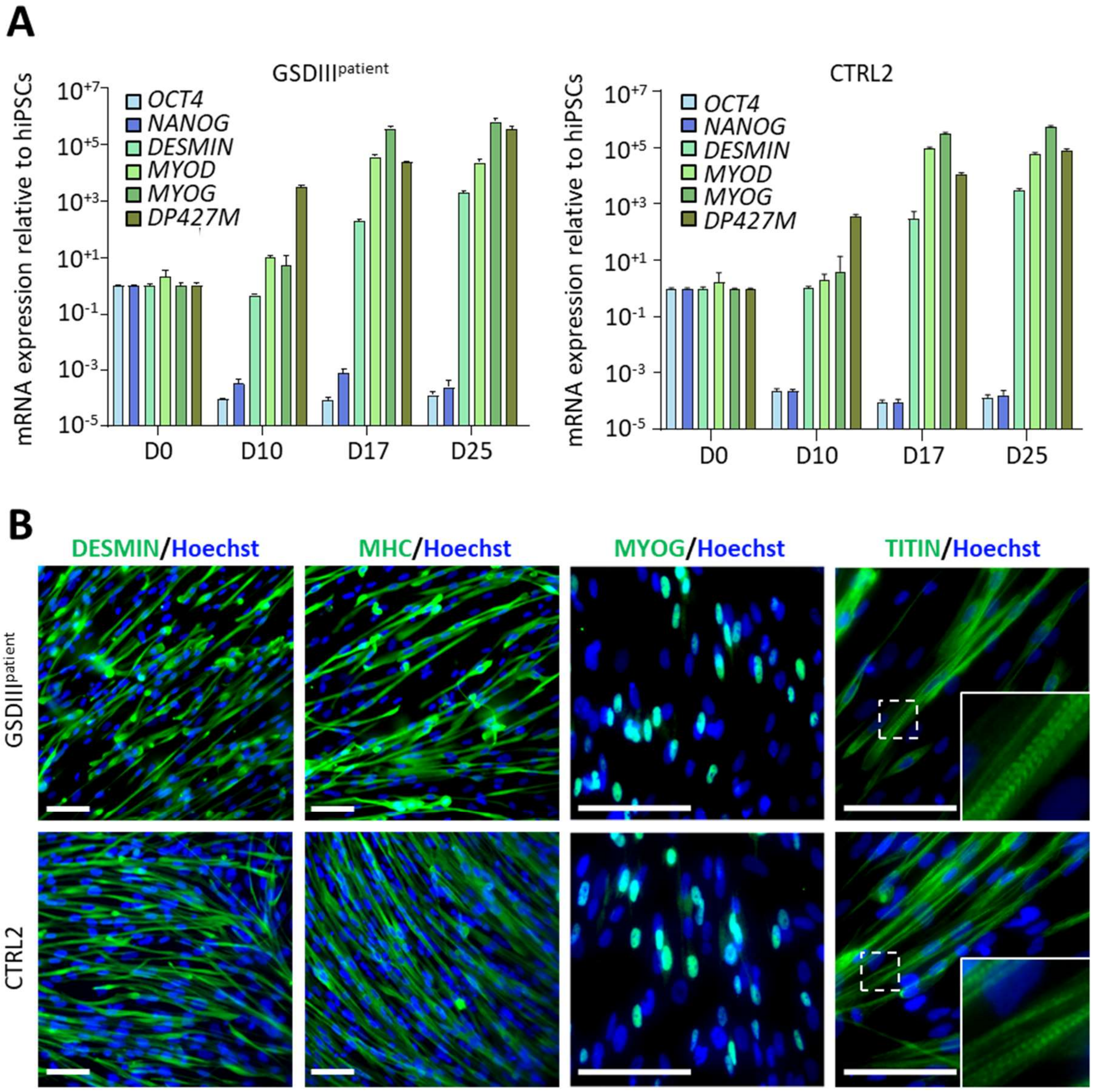

Supplementary Figure S3. Skeletal myogenic differentiation of GSDIII^{patient} and CTRL2 hiPSCs. (A) mRNA expression levels of pluripotency markers (*OCT4, NANOG*) and myogenic markers (*DESMIN, MYOD, MYOG, DP427M*) measured by qPCR in triplicate at day 0, 10, 17 and 25 of differentiation of GSDIII^{patient} and CTRL2 hiPSCs. mRNA expression analyses are normalized to hiPSCs at day 0. (B) Characterization of the expression of desmin, myosin heavy chain (MHC), myogenin (MYOG) and titin by immunostaining in GSDIII^{patient} and CTRL2 skMt (D25). Nuclei are labeled by Hoescht staining (blue). White boxes are magnifications of the titin staining. Scale bar = 50 µm.

Supplementary Figure S4

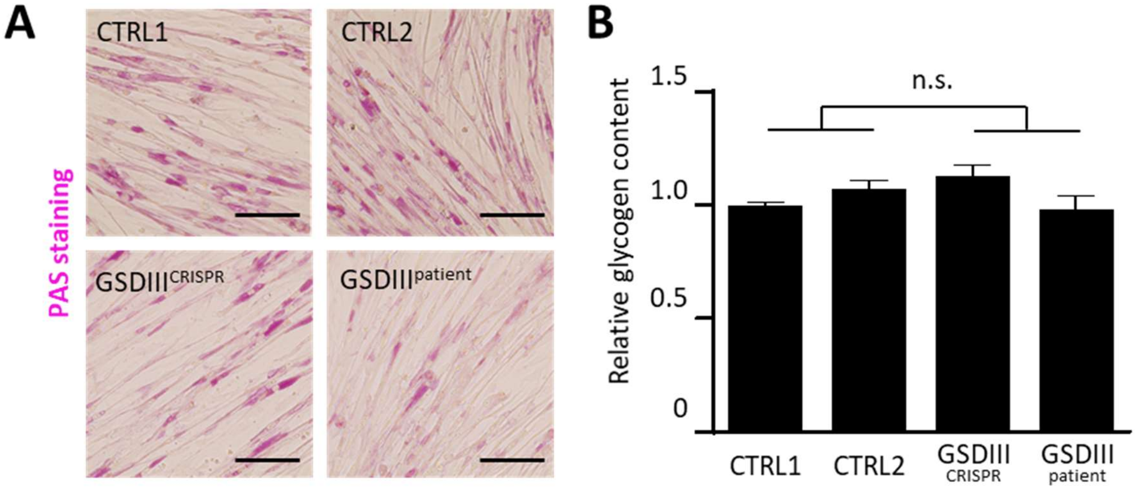

Supplementary Figure S4. Analysis of glycogen content on control and mutated skeletal myotubes without glucose deprivation period. (A) Representative images of Periodic Acid Schiff (PAS) staining on control and mutated skMt. Scale bar = 200 µm. (B) Measurement of glycogen content in control and mutated skMt in triplicate using photometric assay. Glycogen content is expressed as the relative amount of glycogen measured on CTRL1 line.

Supplementary Figure S5

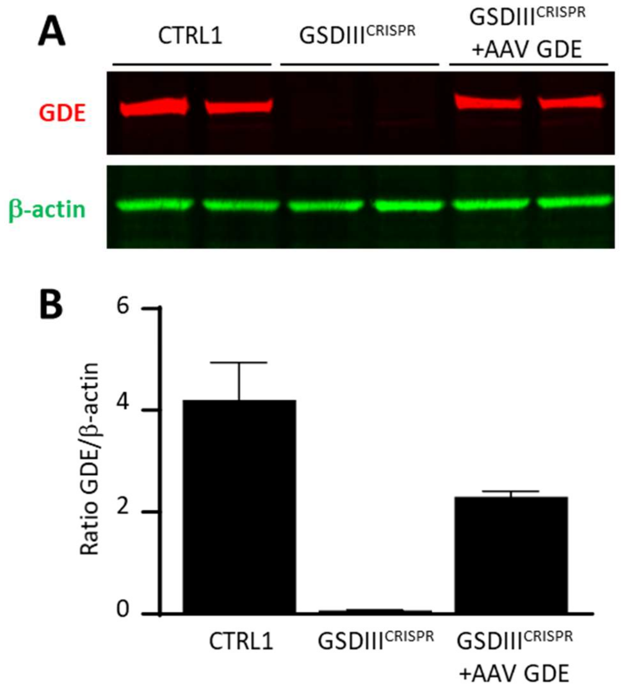

Supplementary Figure S5. Expression of GDE in GSDIII^{CRISPR} skeletal myotubes following transduction with a recombinant AAV expressing the human GDE. (A) Immunoblot analysis of GDE expression (red) in CTRL1 and GSDIII^{CRISPR} skMt treated or not during the myogenic differentiation with a recombinant AAV vector expressing the human full-length GDE. β-actin (green) was a loading control. (B) Quantification of GDE protein expression levels. Values are expressed as the ratio of the signal of the GDE and β-actin bands.

Supplementary Table S1

List of primers (related to Materials and Methods).

Gene	Forward	Reverse
AGL	TGCTTTGCAGGCTACAACATG	AGCCCCTTTCCTTTCTCTTTCT
MGAT5	TAAGTCACACTAGAGGGCCAG	CCAGTGAGAAGTGGCAATGTTC
EML4	CATCCACCTGTCCAGTTGCT	CACTTCATGGCCACATAAACACA
FAM160A1	ATGCCAGGAGCAAACTCTTGA	ATAAGGTGCTCTGGTAGGCAA
ZNF536	TACACAGTGAGGGCCGCATA	GCCAATTGCCTTCTTGTGCT
ADAMTS14	GTGCTTAATGTCCCAGAGCCT	CAAACATGTCATGCACCCCC
LUZP2	AGAACCTTGTGAACCCACCG	ACACCACTGGCTTTTCTGTAA
PRKAR2A-AS1	AGCCCAGGTTCTAGGCTAAG	GGGGCATCTGAGAACAAGTC
CACNA2D3	CACTGCAGACATGGAGGATGA	GGGAGATGGGAGCCAAAGAAC
OCT4	CCTCACTTCACTGCACTGTA	CAGGTTTTCTTTCCCTAGCT
NANOG	CAAAGGCAAACAACCCACTT	TCTGCTGGAGGCTGAGGTAT
DESMIN	ATTGGAGGACCGATTTGCC	TCACCGTCTTCTTGGTATGGA
MYOD	GGGGCTAGGTTCAGCTTTCT	CTACATTTGGGACCGGAGTG
MYOG	TAAGGTGTGTAAGAGGAAGTCG	CCACAGACACATCTTCCACTGT
DP427M	GTGGGAAGAAGTAGAGGACTGT	TCCTGTAGGTCACTGAAGAGGT
GDE transgene	CTGAAGCTGTGGGAGTTCTT	CTCTTGGTCACTCTTCTGTTCTC

Supplementary Table S2

List of the 8 potential off-target sites of the sgRNA.

Off-Target Sequences	Chromosome	Start	End	Locus
TAGGCAAAGAGGCTGAAATGGGG	chr2	135025872	135025894	intron:MGAT5
AAGTCCAATAATCTGCAGTGTGG	chr2	42513452	42513474	exon:EML4
TGGTCCAAAGGTCAGCAATGAGG	chr4	152339828	152339850	intron:FAM160A1
TTGTGCAAGAGTCTACAGTGTGG	chr19	31046678	31046700	intron:ZNF536
GTGTCCCACAGTCTGCAATGAGG	chr10	72490339	72490361	intron:ADAMTS14
TTCTCCAAGAGTCAACAATGTGG	chr11	24970301	24970323	intron:LUZP2
TTCTCCAAGAGTATGCAATAAGG	chr3	48886643	48886665	exon:PRKAR2A-AS1
AAACTCAAGAGTCTGCAATGAAG	chr3	54833606	54833628	intron:CACNA2D3

Supplementary Table S3

List of antibodies (related to Materials and Methods).

Application	Antibodies	Supplier	Reference	Dilution
Immunostaining	NANOG	Cell signaling	4903-s	1:500
	OCT4	Cell signaling	2840-s	1:500
	DESMIN	R&D	AF3844	1:200
	MF20	DSHB	3ea	1:50
	MYOD	Dako	M3512	1:50
	MYOG	DSHB	F5D-c	1:50
	TITIN	US Biological	T5650	1:50
	Donkey anti-rabbit AF555	Invitrogen	A32794	1:1000
	Donkey anti-goat AF488	Invitrogen	A11055	1:1000
	Donkey anti-mouse AF488	Invitrogen	A21202	1:1000
Western Blot	GDE	Agrisera	AS09-454	1:1000
	β-ACTIN	Li-Cor	926-42212	1:4000
	IRDye® 680RD Donkey anti-rabbit	Li-Cor	926-68073	1:5000
	IRDye® 800CW Donkey anti-mouse	Li-Cor	926-32212	1:10000
Flow Cytometry	TRA-1-81 AF647	Biolegend	330706	1:25
	AF647 Isotype control	Biolegend	401618	1:250
	SSEA4 PE	Miltenyi	130-122-914	1:50
	PE Isotype control	Miltenyi	130-113-462	1:50
